# The long noncoding RNA *XIAP-AS1* promotes *XIAP* transcription by *XIAP-AS1* interacting with Sp1 in gastric cancer cells

**DOI:** 10.1371/journal.pone.0182433

**Published:** 2017-08-08

**Authors:** Jun Cai, Dong Wang, Zhi-Gang Bai, Jie Yin, Jun Zhang, Zhong-Tao Zhang

**Affiliations:** Department of General Surgery, Beijing Friendship Hospital Affiliated to Capital Medical University, Beijing, China; University of South Alabama Mitchell Cancer Institute, UNITED STATES

## Abstract

Long noncoding RNAs (lncRNAs) play roles in the tumorigenesis, proliferation and metastasis of tumor cells. Previous studies indicate that the transcription factor Sp1 is responsible for transcription of the *XIAP* gene, but it is unknown whether lncRNAs are involved in *XIAP* transcription. Herein, we identified a novel lncRNA, denoted as *XIAP-AS1*, transcribed from the first intron of the complementary strand of the *XIAP* gene. Using RNA FISH, cell fractionation and qRT-PCR, *XIAP-AS1* was determined to be located primarily in the nucleus. After various *XIAP-AS1* deletion mutants were expressed, RIP assays showed that only the full-length *XIAP-AS1* RNA interacted with Sp1 and thereby participated in *XIAP* transcription. ChIP assays showed that *XIAP-AS1* knockdown decreased the binding of Sp1 to the promoter region of *XIAP*. *XIAP-AS1* knockdown promoted tumor necrosis factor (TNF)-related apoptosis-inducing ligand (TRAIL)-induced apoptosis in gastric tumor cells, as cleaved caspase-3 and caspase-9 was detected. Moreover, in an *in vivo* mouse xenograft model, tumor cell proliferation was inhibited by *XIAP-AS1* knockdown in response to TRAIL administration. In conclusion, our results indicate that *XIAP-AS1* is involved in *XIAP* transcription by interacting with Sp1. Additionally, *XIAP-AS1* is a potential target for TRAIL-induced apoptosis in gastric cancer cells.

## Introduction

Long noncoding RNAs (lncRNAs) are defined as transcripts longer than 200 nucleotides, and they participate in cancer development and metastasis, as well as exert considerable influence on the transcription [1], alternative splicing [2], and translation [3] of target genes. For example, the lncRNA HOTAIR promotes the invasiveness and metastatic potential of human breast cancer cells via recruitment of polycomb repressive complex 2 (PRC2) and induction of H3K27 trimethylation, thereby resulting in altered gene expression [4]. LncRNA MALAT1 is involved in the alternative splicing of target genes by the recruitment of serine/arginine-rich splicing factor 1 (SRSF1) [2]. Yoon. JH. et al. report that lincRNA-p21 selectively lowers the translation of target gene *CTNNB1* and *JUNB* mRNA by its partial complement with target gene mRNAs [3]. The prognostic power of lncRNA signatures has been recently investigated in cancers [5]. With the advancement of in the depth and quality of transcriptome sequencing, increasing number of lncRNAs are found. Although the biological function of some lncRNAs have been disclosed, the function of most lncRNAs remains unknown.

The protein *XIAP* (X-linked inhibitor of apoptosis) inhibits caspase activity and blocks apoptosis. *XIAP* inhibits the activation of caspase-3 and caspase-9 by binding to their BIR2 and BIR3 domains, respectively [6]. Reduced *XIAP* expression sensitizes acute myeloid leukemia cells to TRAIL-induced apoptosis [7], and specific downregulation of Bcl-2 and *XIAP* by RNAi enhances the efficacy of chemotherapeutic agents in MCF-7 human breast cancer cells [8]. Lee et al. reported that the transcription factor Sp1 regulates *XIAP* transcription via binding to the *XIAP* gene promoter [9]. In the present study, we observe a novel lncRNA, *XIAP-AS1*, complementary to the *XIAP* transcript using information regarding the *XIAP* gene obtained from the UCSC genome browser (www.genome.ucsc.edu). However, the function of *XIAP-AS1* is currently still unclear. Additionally, we demonstrate that *XIAP-AS1* participates in regulating *XIAP* transcription by interacting with and enhancing the binding of Sp1 to the *XIAP* gene promoter. Furthermore, *XIAP-AS1* knockdown promotes TRAIL-induced apoptosis in gastric tumor cells, suggesting *XIAP-AS1* as a potential therapeutic target for regulating TRAIL-induced cell death in gastric tumor cells.

## Materials and methods

### Cells and reagents

The gastric cell lines BGC823, SGC7901, MKN28, AGS and MGC803 were maintained in RPMI-1640 medium, and the Kato3 cells were maintained in Dulbecco’s Modified Eagle’s Medium (DMEM) supplemented with 10% FBS. All cells were maintained in an incubator (Shellab, Cornelius, Oregon, USA) at 5% CO_2_ and 37°C. All cell lines were purchased from the Cell Bank of Type Culture Collection of the Chinese Academy of Sciences (Shanghai, China). TRAIL) was purchased from Sigma-Aldrich (St Louis, MO, USA). RPMI-1640, DMEM and fetal bovine serum (FBS) were purchased from HyClone (Logan, Utah, USA). Acrylamide, methylene acrylamide, tris-base, ammonium peroxydisulfate, TEMED, glycine and SDS were purchased from Sangon Biotech, Inc. (Shanghai, China), and the PVDF membrane and chemiluminescence reagents were purchased from Thermo Fisher Scientific, Inc. (Waltham, MA, USA).

### RNA fluorescence *in situ* hybridization (RNA FISH)

*In situ* hybridization was performed as previously described with some modifications [10]. Total RNA was extracted from BGC823 cells using TRIzol (Life Technologies, CA USA), and reverse transcription of the total RNA and PCR of the DNA template for synthesis of the *XIAP*-AS probes were performed according to the manufacturer's instructions (Takara, Dalian, China). The primer sequences for PCR were *XIAP-AS1*: 5'-CATGCCATGGTACCCTGGGAGACAGAATGAAAAGC-3' (forward) and 5'-ACGCGTACGCCACCTGTGTTTCTCAGCCCTTCTCT-3' (reverse). The PCR product was purified, subcloned into the pGM-T vector and confirmed by DNA sequencing. The plasmid was linearized using either *NcoI* or *SalI* (NEB, Beverly, MA, USA) and used as a transcription template for the T7 or Sp6 RNA polymerases (NEB, Beverly, MA, USA) to generate the antisense and sense probes, respectively. The transcription reaction was as follows: 2 μl of biotin-conjugated dNTP mix (Roche, Basel, Switzerland), 2 μl of RNA polymerase, 2 μl of buffer, 1 μg of linearized DNA template, 0.5 μl of RNase inhibitor (NEB, Beverly, MA, USA), 1 μl of 100xBSA and DECP-treated water in a final volume of 20 μl. After 3 μm-thick tissue sections were deparaffinized, dehydrated and heated to 95°C in a microwave oven in 0.01 M citrate buffer (pH 6.0) for 15 min, the slides were treated with 0.3% Triton X-100 in DEPC-treated PBS for 10 min and 10 μg/ml proteinase K for 20 min at 37°C. The tissue sections were incubated with sense or antisense probes overnight at 48°C. After hybridization, the sections were washed three times with 2×SSC and incubated with streptavidin-conjugated Alexa Fluor 488 for 1 h at room temperature at a dilution of 1:100 (Sigma-Aldrich, St Louis, USA). Tissue sections were counterstained with DAPI, and immunofluorescence was observed using an Axio Observer A1 microscope (Carl Zeiss, Germany). The sense probe was used as the negative control.

### Real-time PCR

Total RNA was extracted using TRIzol according to the manufacturer's instructions. The RNA purity and concentration were measured using a NanoDrop2000c spectrophotometer (A260:A280>1.8), and RNA integrity was evaluated by agarose gel electrophoresis combined with ethidium bromide staining. Reverse transcription was performed according to instructions provided by the manufacturer (Takara, Dalian, China). Briefly, contaminating genomic DNA was removed from 1 μg of total RNA with DNase at 42°C for 2 min. Next, 1 μl of the reverse transcription primer mixtures containing oligo (dT) and random primers, 1 μl of reverse transcriptase, 4 μl of 5× reverse transcription buffer and 4 μl of DEPC-treated water were added to the above DNase-treated RNA template in a total reaction volume of 20 μl. The reaction was incubated at 37°C for 15 min and then at 85°C for 5 sec before being stored at 4°C. Real-time PCR was performed with an Applied Biosystems 7500 detection system. For the reaction, 1 μl of cDNA, 12.5 μl of 2× SYBR Green I Master Mix, 10 pmol of the specific forward primer, 10 pmol of the reverse primer, and 0.5 μl of ROX II were combined, and DEPC-treated water was added to a final volume of 25 μl. The reaction parameters consisted of incubation at 95°C for 30 sec, followed by 40 cycles of 95°C for 5 sec and 60°C for 34 sec. The relative target gene quantification was calculated using the 2^-ΔΔCt^ method [11], where ΔΔCt = [Ct (treated group) target gene – Ct (treated group) internal control] – [Ct (control group) target gene – Ct (control group) internal control]. β-Actin was used as the internal control. The primer sequences for real-time PCR were as follows: *XIAP-AS1*: 5'-TACCCTGGGAGACAGAATGAAAAGC-3' (forward) and 5'-CACCTGTGTTTCTCAGCCCTTCTCT-3' (reverse); *XIAP*: 5’-ATGACAGGGCTGGAAGTGACC-3’ (forward) and 5’-ACTATGTCCCAGTCAGGGCTCT-3’ (reverse); *β-actin*: 5’-CTTAGTTGCGTTACACCCTTTCTTG-3’ (forward) and 5’-CTGTCACCTTCACCGTTCCAGTTT-3’ (reverse). The primers were synthesized by Sangon Biotech Company (Shanghai, China).

### ChIP-qPCR

ChIP-qPCR was performed as previously described [12] with some modifications. BGC823 cells stably infected with lentiviruses expressing either an *XIAP-AS1* shRNA (pLenti-*XIAP-AS1*-shRNA) or a scrambled shRNA (pLenti-Scrambled-shRNA) were treated with 4% formaldehyde at room temperature for 15 min. Glycine was added to a final concentration of 125 mM to halt the cross-linking reaction. After being washed twice with ice-cold PBS, the cells were collected and resuspended in 600 μl of cell lysis buffer (150 mM NaCl, 50 mM Tris-HCl (pH 7.5), 5 mM EDTA, 0.5% NP-40, 1.0% Triton X-100) containing proteinase inhibitors and centrifuged at 12,000 g for 1 min at 4°C. The pellets were then resuspended in lysis buffer and sonicated for 10 pulses at 20 sec per pulse, with 30 sec on ice between the pulses, and the resulting homogenates were centrifuged at 12,000 g for 10 min at 4°C. The supernatants were separately incubated with rabbit anti-Sp1 antibody, rabbit anti-AP-1 antibody or normal rabbit IgG antibody (Santa Cruz Biotechnology, CA, USA) overnight at 4°C. The next day, the immunocomplexes were incubated with Dynabeads Protein G (Life Technologies, CA, USA) for 2 h at 4°C and recovered using magnets. The chromatin–antibody–Dynabead complexes were washed three times with PBS and treated with 0.25 mg/ml proteinase K (Sigma-Aldrich, St Louis, MO, USA) at 37°C for 12 h. DNA was extracted, and PCR was performed. The PCR primers for the *XIAP* promoter were 5'-GGAGGGGGGTAAGATTTGAGAGGTA-3' (forward) and 5'–TTACAGTCATTAGGTGGGACGCTTT -3' (reverse).

### RNA immunoprecipitation (RIP)

The RIP experiments were conducted using the Magna RIP^™^ RNA-Binding Protein Immunoprecipitation Kit (Millipore, MA, USA) according to the manufacturer's instructions. Briefly, the rabbit anti-Sp1 antibody, rabbit anti-AP-1 antibody or rabbit normal IgG antibody was incubated with the cellular extracts overnight at 4°C (Santa Cruz Biotechnology, CA, USA), and 10% of the cellular extracts was used as the input control and stored at -80°C. The next day, Dynabeads Protein G (Life technology, CA, USA) were added to the complex, the mixture was incubated for 2 h at 4°C and the immunoprecipitates were recovered using magnets. RNA molecules from the immunoprecipitates and from the 10% of the input control were extracted using TRIzol, and reverse transcription was performed. Gene specific primers for *XIAP-AS1* were then used for real-time PCR.

### Construction and transfection of full-length and deletion-mutant *XIAP-AS1* expression vectors

Full-length *XIAP-AS1* and deletion mutants were synthesized and subcloned into pcDNA3.1 by Beijing GENEWIZ Inc. (Beijing, China), and the cloned sequences were confirmed by sequencing. Transfection of *XIAP-AS1* and the deletion mutants was performed using the lipofectamine 3000 reagent (Life Technologies, CA, USA). In brief, MNK28 cells were inoculated in 60-mm dishes and cultured for 24 h. Then, 5 μg of vector complexed with 7.5 μl of lipofectamine 3000 was transfected into MNK28 cells. Cells were incubated for an additional 48 h, and RIP assays for Sp1 or IgG were then performed. Relative enrichment of *XIAP*-AS and of the deletions was determined using qRT-PCR. The primer sequences for *XIAP*-AS and the deletion mutants were as follows: 5'-AGACGGACTCTTGCTGTGTCGCCCA-3' (forward), 5'-CCCAGGCTCAGGCAGTCCTTCCACC-3' (reverse). SiRNA transfection was performed as previously described [13] with slight modifications. Briefly, *XIAP* siRNA or scrambled siRNA was transfected into BGC823 cells using lipofectamine 3000. After 48 h, the treated cells were collected. The sequences for the *XIAP* siRNA were 5'-GGAGAUACCGUGCGGUGC-3' (sense) and 5'-GCACCGCACGGUAUCUCCdtdt-3' (antisense). The negative control siRNA sequence was 5'-UUCUCCGAACGUGUCACGdtdt-3' (sense) and 5'-CGUGACACGUUCGGAGAA-3' (antisense).

### Western blot

The *XIAP* protein was measured with mouse anti-*XIAP* antibody (Santa Cruz, CA, USA) by western blot. The cells were collected, washed with prechilled PBS, and lysed in RIPA buffer (Millipore, MA, USA) before the protein concentrations were measured with a BCA kit (Thermo Fisher Scientific, Waltham, MA, USA). Western blotting was performed as previously reported [10]. The primary antibodies, rabbit anti-XIAP, anti-caspase-3, anti-caspase-9, and mouse anti-β-actin (1:1000 dilution), were individually added to the membranes and incubated overnight at 4°C. The next day, the membranes were repeatedly washed in 0.1% TBST (tris-buffered saline and Tween-20) and subsequently incubated with a secondary HRP-conjugated goat anti-rabbit IgG antibody or an HRP-conjugated goat anti-mouse IgG antibody (1:4,000 dilution) at room temperature. All the primary and secondary antibodies were purchased from Santa Cruz Biotechnology (Santa Cruz, CA, USA).

### Cytoplasmic and nuclear extract isolation

Approximately 1x10^7^ BGC823 cells were resuspended in 500 μl of cytoplasmic extract buffer with NP40 (10 mM HEPES, 60 mM KCl, 1 mM EDTA, 0.075% (v/v) NP40, 1 mM DTT and 1 mM PMSF, pH 7.6) and incubated on ice for 3 min. The extracts were subjected to centrifugation at 1500 g for 4 min, and the cytoplasmic supernatants were then transferred to a clean tube. The nuclear pellets were resuspended in 100 μl of nuclear extraction buffer (20 mM tris-HCl, 420 mM NaCl, 1.5 mM MgCl2, 0.2 mM EDTA, 1 mM PMSF and 25% (v/v) glycerol, pH 8.0) and vortexed for 1 h at 4°C. The nuclear extracts were then collected by centrifugation at 12,000 g for 10 min. RNA derived from the cytoplasmic and nuclear extracts was extracted using TRIzol (Life Technologies, CA, USA), and *XIAP-AS1* was verified using qRT-PCR. *U6* RNA and *GAPDH* mRNA were selected as the nuclear and cytoplasmic control transcripts, respectively. The primers for *U6* RNA were 5'-CTCGCTTCGGCAGCACA-3' (sense) and 5'-AACGCTTCACGAATTTGCGT-3' (antisense). The primers for *GAPDH* mRNA were 5'-AGAACATCATCCCTGCCTCTACTGG-3' (sense) and 5'-CCTGCTTCACCACCTTCTTGATGTC-3' (antisense).

### Production of lentivirus encoding shRNAs and construction of the BGC823 *XIAP-AS1* shRNA stable cell lines

The pLenti-*XIAP-AS1*-shRNAand pLenti-Scrambled-shRNA lentiviruses were constructed by the Shanghai Hanbio Biotechnology Company (Shanghai, China). All the shRNAs were comprised of a short sense strand of 19 nucleotides followed by a loop of 9 nucleotides and the analogous antisense strand. The shRNAs were subsequently subcloned into the pLenti-super vector. Lentivirus packaging was performed by the Shanghai Hanbio Biotechnology Company. The *XIAP-AS1* shRNA sequences were 5′-GATCCAAAAGAGAGAGAGAGGGATTCGAGAATCCCTCTC TCTCTCTTTTTTTTTG-3′ (sense) and 5′-AATTCAAAAA AAAAG AGAGAGAGA GGGATTCGAGAATCCCTCTCTCTCTCTTTTG-3′ (antisense). The negative control shRNA sequence was 5'-GATCCTTCTCCGAACGTGTCACGTCTCGAGAC GTGACACGTTCGGAGAATTTTTG-3' (sense) and 5'-AATTCAAAAATTCTCCG AACGTGTCACGTCTCGAGACGTGACACGTTCGGAGAAG-3' (antisense). BGC823 cells (1x10^5^) were inoculated onto a 6-well plate 24 h prior to the lentiviral transfection. Lentivirus (5 μl) was individually added to the wells and mixed gently. The next day, 6 μg/ml polybrene (Sigma-Aldrich, St Louis, USA) in 1 ml of fresh culture medium was added and incubated for 4 h, and then 1 ml of fresh culture medium was added again. After infection for 24 h, fresh culture medium with 500 ng/ml puromycin was added, and the cell clones were selected after two weeks. *XIAP* expression was detected by qRT-PCR.

### Immunohistochemistry (IHC)

The tumor tissues used for IHC were obtained from BGC823 shRNA-*XIAP-AS1* or shScramble cells. The 4 μm paraffin-embedded sections were deparaffinized, rehydrated. The procedures of IHC for XIAP were performed according to the proctocol [14]. The sections were incubated with mouse anti-XIAP antibody (Santa Cruz, CA, USA) diluted as 1:200 overnight at 4°C. Normal rabbit IgG antibody used as negative control. The sections were developed with 3, 3′-diaminobenzidine tetrahydrochloride (DAB) after being incubated with HRP-conjugated goat anti-mouse secondary antibody (Zhongshan Goldbridge Biotechnology, China) and then the slides were counterstained using Mayer’s hematoxylin.

### Measurement of cell apoptosis

Apoptosis was measured by Annexin V staining using a Cell Apoptosis Detection kit (Beyotime Biotechnology, Jiangsu, China) according to the manufacturer’s instructions, and the percentage of apoptotic cells was determined by flow cytometry (BDAccuri C6, BD Biosciences, Franklin Lakes, NJ, USA). Briefly, BGC823 stable cells infected with either the pLenti-shRNA-*XIAP-AS1* or pLenti-shScramble cells were treated with 100 ng/ml TRAIL for 24 h, and the cells were then trypsinized and washed with cold PBS. The cells were harvested by centrifugation at 1,000 g for 5 min, and the pellet, containing approximately 1.0 × 10^5^ cells, was resuspended in 195 μl of binding buffer before being incubated with 5 μl of Annexin V-FITC and 10 μl of PI (Sigma-Aldrich, St Louis, USA) at room temperature for 10 min in the dark. The cells were subsequently collected and washed with cold PBS, and the percentage of apoptotic cells was analyzed using flow cytometry.

### Mice xenografts

All animal experiments were approved by the Health Sciences Animal Policy and Welfare Committee of Beijing Friendship Hospital Affiliated to Capital Medical University. In the present study, six-week-old female BALB/c nude mice (nu/nu; n = 6) (Beijing Weitong Lihua Experimental Animals Company, China) were anesthetized with an isoflurane/propylene glycol mixture, and BGC823 stable cell lines with pLenti-shRNA-*XIAP-AS1* or pLenti-shScramble cells were subcutaneously injected into the right flank of each mouse (1.0 × 10^6^ cells in 100 μl of RPMI 1640 medium per flank). After one week, TRAIL at a dose of 100 μg/mouse was injected directly into the tumor at multiple sites. Every two days, the above treatment was repeated. The tumor sizes were assessed weekly by measuring two dimensions, [length (a)] and [width (b)], and the tumor volumes were calculated as V = ab^2^/2, [15]. The mice were euthanized by CO_2_ inhalation after 30 days, and the tumors were collected and weighed. Mice were handled according to the guidelines of the Health Sciences Animal Policy and Welfare Committee of Beijing Friendship Hospital Affiliated to Capital Medical University. Total protein was extracted from each tumor, and the levels of cleaved caspase-3 and caspase-9 were determined by western blot.

### Statistical analysis

Statistical analysis was performed using SPSS 11.0. Data are presented as the mean ± standard error (SE) using two way analysis of variance (ANOVA). Differences were considered statistically significant when *p* < 0.05.

## Results

### *XIAP-AS1* expression and location in gastric cancer cells

**Fig 1:**
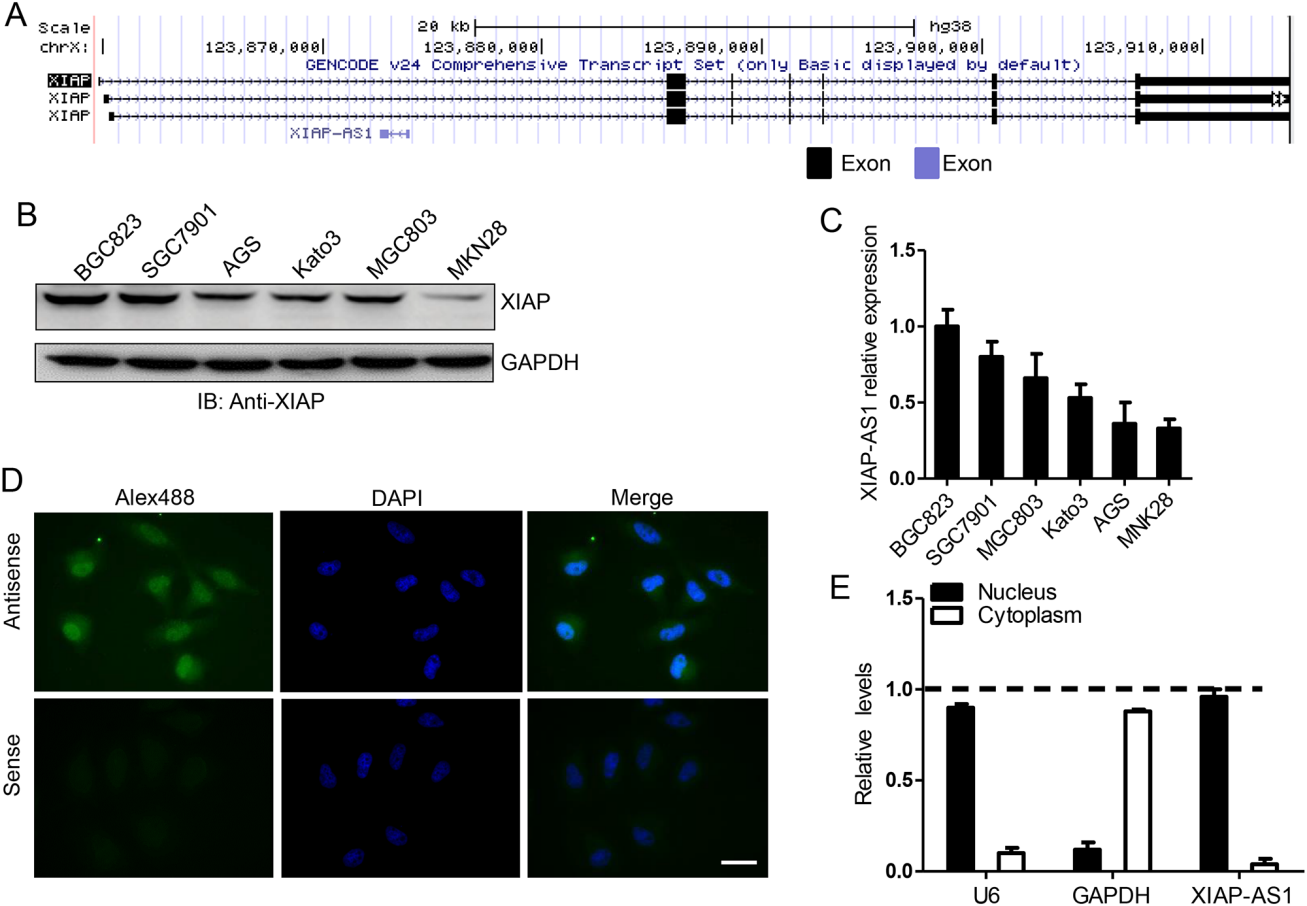
*XIAP-AS1* and *XIAP* expression are correlated and localized in the nucleus. *XIAP-AS1* and *XIAP* gene structure analysis provided by the UCSC Genome Browser (http://genome.ucsc.edu/). The black and light blue boxes represent exons. **(B)** Western blot analysis of *XIAP* expression. **(C)** QRT-PCR analysis of *XIAP-AS1* expression in various gastric cancer cell lines. **(D)** RNA FISH showing the localization of *XIAP-AS1* to the nucleus. BGC823 cells were incubated with *XIAP-AS1* antisense or sense probes conjugated to biotin, and the biotin signal was detected with Alexa Fluor 488-conjugated streptavidin. The sense probe was used as a negative control. After DAPI staining, fluorescence was observed under a fluorescence microscope. Scale bar: 20x magnification. **(E)** The cytoplasms and nuclei of BGC823 cells were isolated, and *XIAP-AS1* was measured by qRT-PCR. *U6* RNA was selected as the nuclear control, and *GAPDH* mRNA was used as the cytoplasmic control transcript.

According to information regarding the *XIAP* gene, *XIAP-AS1* is transcribed from the complementary strand (Fig 1A). We initially determined *XIAP-AS1* and *XIAP* expression using qRT-PCR. *XIAP-AS1* was expressed at various levels in all the gastric cancer cell lines, and *XIAP-AS1* expression was positively correlated with *XIAP* expression (Fig 1B and 1C). Also, the levels of *XIAP-AS1* in stomach adenocarcinoma (n = 285) and normal tissues (n = 33) derived from TCGA were analyzed and the results showed that *XIAP-AS1* was highly expressed in stomach adenocarcinoma (S1A Fig). However, there was no significant correlation between the levels of *XIAP-AS1* and survival probability (*P* = 0.07) (S1B Fig). Subsequently, we probed the location of *XIAP-AS1* in gastric cancer cells using RNA FISH and determined that *XIAP-AS1* was mostly distributed in the nucleus (Fig 1D). This nuclear location was further confirmed by isolating the cytoplasmic and nuclear extracts followed by qRT-PCR *XIAP-AS1* (Fig 1E). The nuclear location of *XIAP-AS1* suggests that *XIAP-AS1* may play a role in *XIAP* gene transcription and/or mRNA precursor alternative splicing.

### *XIAP-AS1* interacts with Sp1 to promote its binding to the *XIAP* gene promoter and enhance *XIAP* transcription

**Fig 2:**
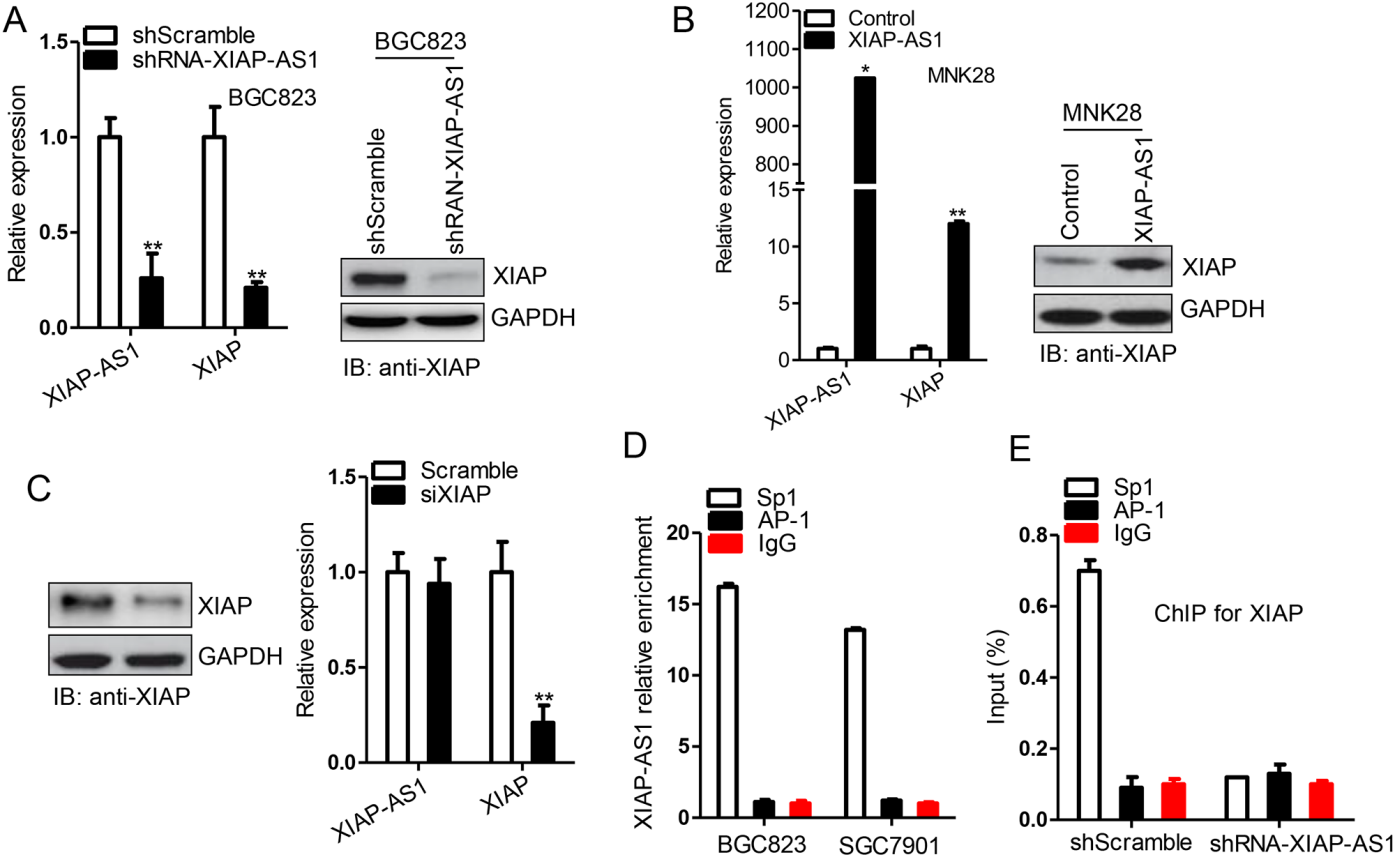
*XIAP-AS1* enhances *XIAP* transcription. **(A)** *XIAP-AS1* and *XIAP* expression was detected with qRT-PCR and western blot, respectively, in the BGC823 shRNA-*XIAP-AS1* or shScramble stable cell lines. **(B)** The *XIAP-AS1* expression vector and the control vector were transfected into MNK28 cells, and *XIAP-AS1* and *XIAP* expression was detected after 48 h. The data are presented as the median ± standard error (SE). *P≤0.05, **P≤0.01. **(C)** siRNA against *XIAP* and scrambled siRNA were transfected into BGC823 cells, and *XIAP-AS1* expression was detected by qRT-PCR after 48 h. Data are presented as the median ± standard error (SE). *P≤0.05, **P≤0.01. **(D)** BGC823 or SGC7901 cells were collected and 10% of extracts was used as input and the rest in triplicate was used in RNA immunoprecipitation for Sp1, AP-1 and rabbit normal IgG. The co-immunoprecipitated RNA and input RNA was extracted and the fold enrichment of *XIAP-AS1* was determined with qRT-PCR. AP-1 and rabbit normal IgG used as control. **(E)** Extracts derived from the BGC823 shRNA-*XIAP-AS1* and shScramble stable cell lines were incubated with rabbit anti-Sp1, rabbit anti-AP-1 or rabbit normal IgG antibodies, and the immune complexes were precipitated with magnetic beads conjugated to Protein G. A total of 10% of the whole cell extract was used as the internal control. DNA was extracted from the immunoprecipitated complexes, and expression of the *XIAP* promoter was determined by real-time PCR. Data are presented as the median ± standard error (SE).

Since *XIAP-AS1* is derived from the strand complementary to *XIAP*, we speculated *XIAP-AS1* to be a potential candidate for regulating *XIAP* expression. To test this hypothesis, we stably overexpressed *XIAP-AS1* in MNK28 cells and stably knocked down *XIAP-AS1* in BGC823 cells. We observed that *XIAP-AS1* overexpression has led to an increase in *XIAP* expression, whereas *XIAP-AS1* knockdown led to a corresponding decrease of *XIAP* expression (Fig 2A and 2B), suggesting the involvement of *XIAP-AS1* in enhancing *XIAP* transcription. Next, *XIAP* was knocked down, which did not alter the *XIAP-AS1* transcript levels (Fig 2C), further indicating that *XIAP-AS1* participates in the regulation of *XIAP* transcription.

Sp1, a known transcription factor, has been shown to mediate *XIAP* transcription. Thus, we speculated that *XIAP-AS1* interacts with Sp1 to regulate *XIAP* gene transcription. Therefore, to ascertain an interaction between *XIAP-AS1* and Sp1, we performed RIP assays in BGC823 and SGC7901 cells and probed for Sp1. The RIP assay suggested an interaction between *XIAP-AS1* and Sp1 (Fig 2D), consistent with our expectations. ChIP assays comparing the BGC823 shRNA-Scramble and BGC823 shRNA-*XIAP-AS1* cell lines showed decreased Sp1 binding to the *XIAP* promoter in the BGC823 shRNA-*XIAP-AS1* cells (Fig 2E), suggesting that *XIAP-AS1* increases Sp1 binding to the *XIAP* gene promoter to enhance *XIAP* transcription.

### Full-length *XIAP-AS1* is required for *XIAP-AS1* and Sp1 interaction

**Fig 3:**
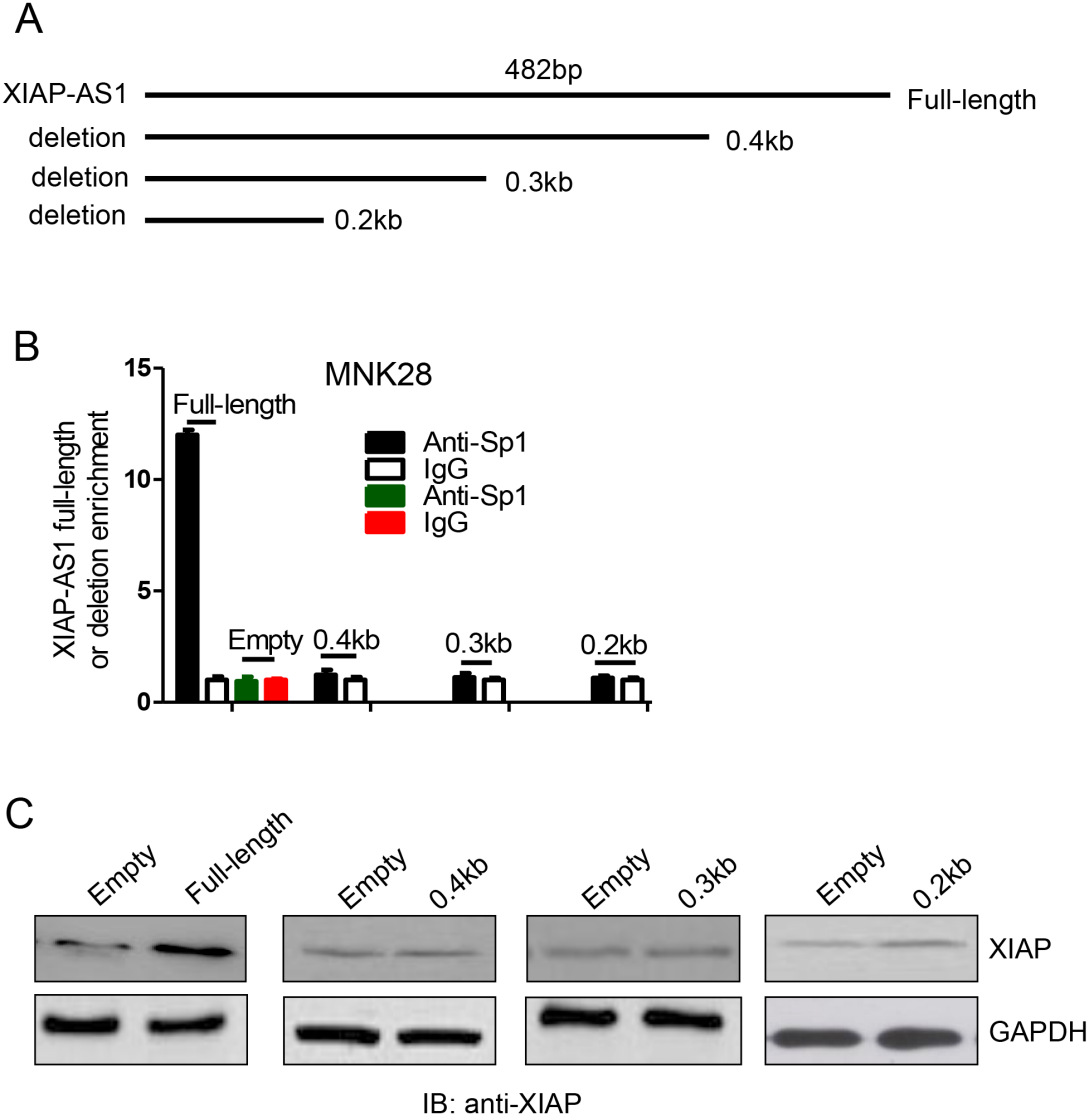
Full-length *XIAP-AS1* is required for interaction with Sp1. **(A)** Schematic representation of the full-length and truncated *XIAP-AS1*. **(B)** *XIAP-AS1* and the deletions were individually transfected into MNK28 cells. After 48 h, extracts derived from the treated cells were incubated with a rabbit anti-Sp1 or a normal rabbit IgG antibody. The Sp1 complexes were subjected to pulldown using magnetic beads conjugated to Protein G. After RNA was extracted, the bound full-length and mutant *XIAP-AS1* was measured by qRT-PCR. Empty vector was used as the negative control. Data are presented as the median ± SE. **(C)** Protein from the above treated cells was extracted, and XIAP was detected with mouse anti-XIAP antibody by western blot.

To identify the region of *XIAP-AS1* required for its interaction *XIAP-AS1* with Sp1, we performed RIP assays for Sp1 using various *XIAP-AS1* deletion mutants. In contrast to full-length *XIAP-AS1*, none of the *XIAP-AS1* deletion mutants pulled down SP1 (Fig 3A and 3B). Additionally, none of the *XIAP-AS1* deletions had an effect on *XIAP* expression, whereas full-length *XIAP-AS1* prompted *XIAP* up-regulation (Fig 3C). These results show that full-length *XIAP-AS1* is indispensable for the interaction between *XIAP-AS1* and Sp1.

### *XIAP-AS1* knockdown enhances TRAIL-induced apoptosis *via* caspase activation

**Fig 4:**
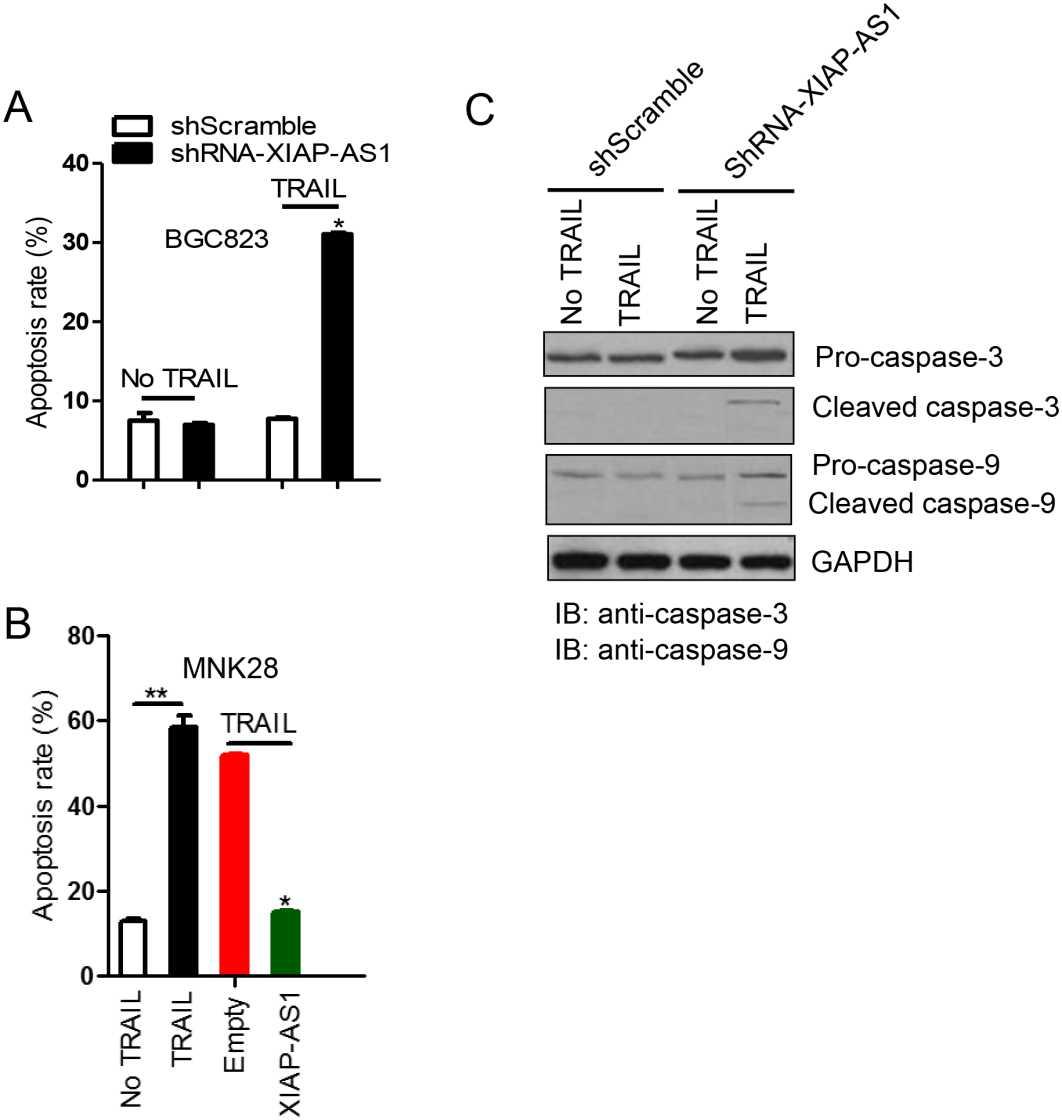
*XIAP-AS1* knockdown promotes TRAIL-induced apoptosis. **(A)** BGC823 shRNA-*XIAP-AS1* and shScramble cells were treated with TRAIL at a final concentration 100 ng/ml for 24 h, and the percent of apoptotic cells was determined by flow cytometry with Annexin V staining. **(B)** Cleaved caspase-3 and caspase-9 were detected by western blot. **(C)** MNK28 cells were transfected with either the *XIAP-AS1* expression vector or an empty vector and then treated with TRAIL for 24 h. The percentage of apoptotic cells was determined using flow cytometry with Annexin V staining.

To examine the effect of *XIAP-AS1* knockdown on TRAIL-induced apoptosis, we quantified the percentage of apoptotic cells using flow cytometry with Annexin V staining after either the BGC823 shScramble or the shRNA-*XIAP-AS1* cells exposed to TRAIL. We observed no obvious apoptosis in either the shScramble cells or the shRNA-*XIAP-AS1* cells in the absence of TRAIL treatment. However, upon treatment with TRAIL, apoptosis in the shRNA-*XIAP-AS1* cells increased (Fig 4A and S2A Fig). Conversely, TRAIL induced apoptosis of MNK28 cell and overexpression of *XIAP-AS1* in MNK28 cells inhibited TRAIL-induced apoptosis (Fig 4B and S2B Fig). To examine whether *XIAP-AS1* knockdown enhanced TRAIL-induced cell apoptosis *via* caspase activation, we measured caspase cleavage in BGC823 shRNA-*XIAP-AS1* cells treated with TRAIL. TRAIL treatment resulted in caspase (caspase-3 and caspase-9) cleavage after *XIAP-AS1* knockdown *XIAP-AS1*. In response to *XIAP-AS1* knockdown, the 32 kDa pro-caspase-3 protein was cleaved to a 20 kDa intermediate and the active p17 subunit (Fig 4C). Levels of the precursor protein pro-caspase-9 were also subjected to proteolysis in response to *XIAP-AS1* knockdown. These results suggest that *XIAP-AS1* knockdown enhances TRAIL-induced apoptosis *via* promotion of caspase protein activation.

### *XIAP-AS1* knockdown promotes TRAIL-induced apoptosis and inhibits the proliferation of gastric cancer cells *in vivo*

**Fig 5:**
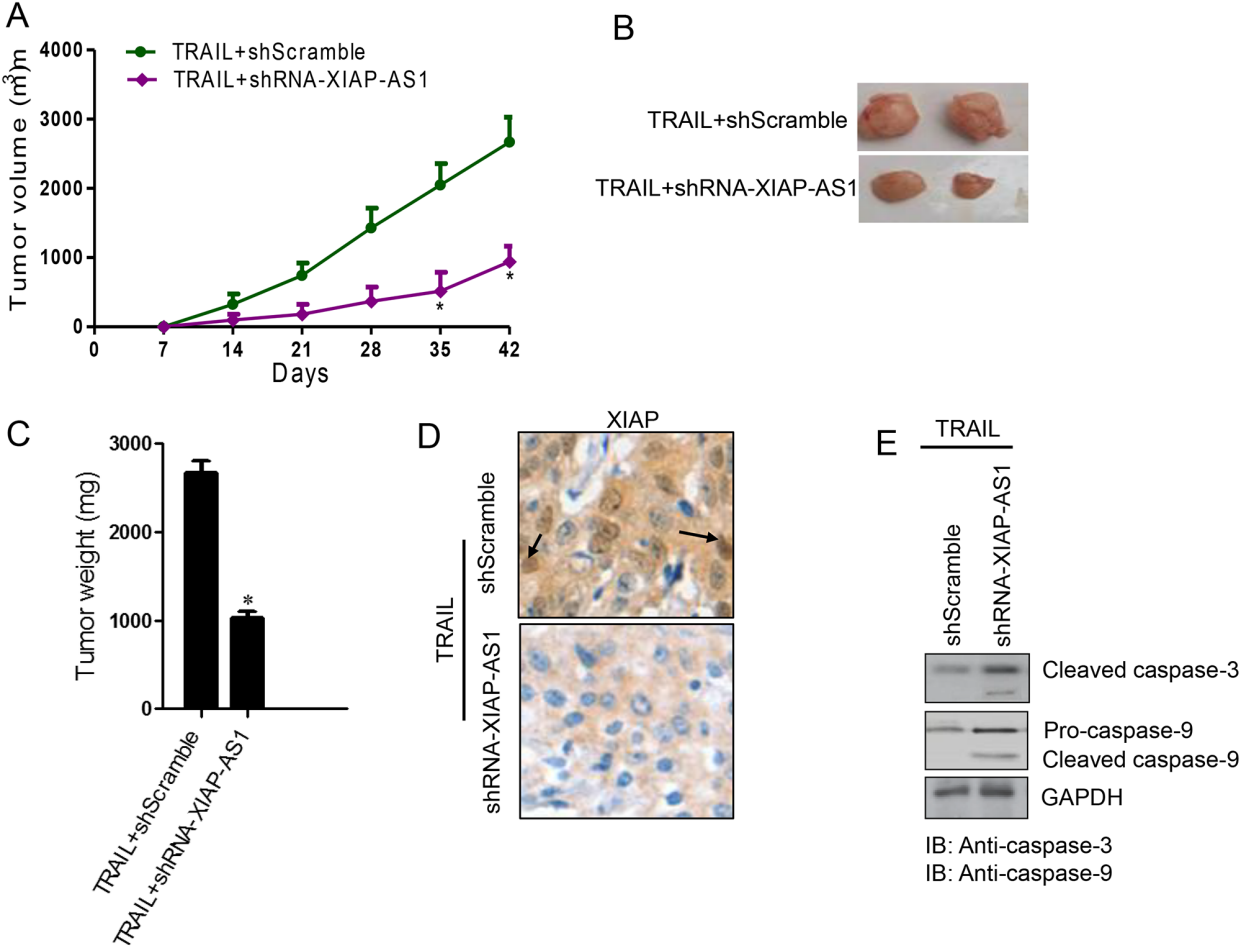
TRAIL inhibits the proliferation of *XIAP-AS1* gastric cancer cells in which *XIAP-AS1* is knocked down and activates caspase-3 *in vivo*. BGC823 shRNA-*XIAP-AS1* cells or the shScramble cells were subcutaneously injected into the right flanks of the mice at 1.0×10^6^ cells/mouse (n = 6). After one week, TRAIL at a dose of 100 μg/mouse was injected into the tumor tissues. **(A)** The tumor size was determined weekly by measuring two dimensions, [length (a)] and [width (b)], and the volume was calculated as V = ab^2^/2. **(B** and **C)** The mice were euthanized after 30 days, and the tumors were collected and weighed. **(D)** XIAP in tumors from BGC823 shRNA-*XIAP-AS1* or shScramble cells was detected with mouse anti-XIAP antibody by immunohistochemistry. **(E)** Total protein was extracted from the tumors, and caspase-3 and caspase-9 were detected by western blot.

To investigate whether *XIAP-AS1* knockdown promotes TRAIL-induced apoptosis and thereby inhibits the proliferation of cancer cells *in vivo*, we intratumorally administered TRAIL to tumors resulting from BGC823 shScramble and shRNA-*XIAP-AS1* cells. Visually, the mean tumor volumes of the shRNA-*XIAP-AS1* cells were smaller than those of the shScramble cells (Fig 5A). To confirm this observed difference, the whole tumor tissues were weighed after the mice were sacrificed. The weight of the tumor tissues from the shRNA-*XIAP-AS1* cells were lighter than those of the controls (Fig 5B and 5C). Immunohistochemistry staining for *XIAP* showed that *XIAP* expression was down-regulated in shRNA-*XIAP-AS1* tumor tissues, compared to shScramble (Fig 5D). Next, we measured the expression levels of cleaved caspase-3 and caspase-9 in the *XIAP-AS1* knockdown and control tumor tissues. Western blot analysis for caspase-3 and caspase-9 demonstrated that pro-caspase-3 and pro-caspase-9 were subjected to more proteolytic processing in the shRNA-*XIAP-AS1* tumor tissues than in the control tumor tissues (Fig 5E). Our results further indicate that *XIAP-AS1* knockdown promotes cellular apoptosis induced by TRAIL *in vivo*.

## Discussion

LncRNAs regulate diverse cellular processes and implicate in the invasion and metastasis of cancer cells. LncRNAs are characterized into the cytoplasmic and nuclear lncRNAs according to the cellular distribution of lncRNAs [16]. The cytoplasmic lncRNAs may participate in the protein translation [3] and the nuclear lncRNAs may exert effects on the transcription and/or alternative splicing of putative target genes [1, 2]. In this study, we show that *XIAP-AS1*, derived from the complementary strand of the *XIAP* gene, plays a role in the transcription of *XIAP* by interacting with Sp1.

TRAIL, a member of the tumor necrosis factor family of cytokines, induces apoptosis and preferentially kills tumor cells but is not toxic to normal cells. Thus, interest in the potential application of TRAIL has emerged in clinical cancer therapy [17]. TRAIL induces apoptosis *via* both the mitochondrial-dependent pathway and the death receptor pathway in tumor cells [18]. *XIAP* has been shown to regulate the response of cancer cells to chemotherapy and radiotherapy *in vitro* [19], and *XIAP* up-regulation has been observed in human prostate, lung and acute/chronic leukemia tumor cells [6]. Because *XIAP* inhibits apoptosis by binding and inhibiting effector caspases, targeting *XIAP* represents a promising strategy for a wide spectrum of malignancies [20]. Most human pancreatic cancers are resistant to TRAIL treatment. Small molecule *XIAP* inhibitors have been shown to synergize with TRAIL to induce apoptosis and inhibit the long-term clonogenic survival of pancreatic carcinoma cells [21]. Downregulation of *XIAP* induces increased caspase-3 cleavage and TRAIL-induced apoptosis [22].

In the present study, the results of RIP and ChIP assays demonstrated that *XIAP-AS1* interacted with Sp1 to regulate *XIAP* transcription (Fig 2). Down-regulating *XIAP-AS1* resulted in *XIAP* knockdown and subsequent caspase-9 activation, which is required for downstream caspase activation events, including caspase-3 activation (Fig 4). Caspase-3, in turn, has been shown to activate caspase-9 through a feedback amplification loop in which caspase-3 cleaves the N-terminal region of caspase-9 and inactivates *XIAP* itself [23]. It is possible for *XIAP*-AS to participate in the resistance of gastric cancer cells to TRAIL-inducing apoptosis by the regulation of *XIAP* transcription. *XIAP-AS1* is highly expressed in BGC823 cells and BGC823 cells treated with TRAIL have no significant effect on cell apoptosis, compared to control. However, *XIAP-AS1* knockdown resulted in the knockdown of *XIAP* and promoted BGC823 cell apoptosis induced by TRAIL (Figs 1C and 4A), indicating that *XIAP-AS1* was implicated in the resistance of BGC823 cells to TRAIL-inducing apoptosis. Also, it is possible for various gastric cancer cell lines to have different response to TRAIL-inducing apoptosis. E.g. *XIAP-AS1* in low expression in MNK28 cells and MNK28 cells were very sensitive to TRAIL-inducing apoptosis. However, *XIAP-AS1* up-regulation inhibited the apoptosis induced by TRAIL (Figs 1C and 4B), which further showed *XIAP-AS1* involvement in the resistance of gastric cancer cells to TRAIL. Therefore, targeting *XIAP-AS1* is likely a useful therapeutic strategy for TRAIL-induced tumor cell apoptosis.

In summary, we show that *XIAP*-AS enhances *XIAP* transcription *via* interacting with Sp1 and that *XIAP*-AS may be a potential therapeutic target for gastric cancer.

## Supporting information

S1 Fig XIAP-AS1 expression levels and the survival probability of patients with stomach adenocarcinoma were analyzed. **(A)** XIAP-AS1 expression levels, down-load from TCGA, in stomach adenocarcinoma (n = 285) and normal tissues (n = 33) were analyzed. **(B)** The survival probability of patients with stomach adenocarcinoma with XIAP-AS1 high (n = 76) or low (n = 209) expression were analyzed.
(DOCX)

S2 Fig XIAP-AS1 knockdown promotes TRAIL-induced apoptosis, whereas XIAP-AS1 up-regulation inhibits the apoptosis induced by TRAIL. **(A)** BGC823 shRNA-XIAP-AS1 or shScramble cells were subjected to no TRAIL or TRAIL treatment at a final concentration 100 ng/ml for 24 h and then the percent of apoptotic cells was determined by flow cytometry for Annexin V staining. **(B)** MNK28 cells were treated without or with TRAIL or the cells were transfected with the XIAP-AS1 expression or empty vector and subsequently treated with TRAIL for 24 h, then the percentage of apoptotic cells was determined using flow cytometry for Annexin V staining.
(DOCX)
