# Supplementary material for: The long noncoding RNA XIAP-AS1 promotes XIAP transcription by XIAP-AS1 interacting with Sp1 in gastric cancer cells
Source: PLoS One. 2017 Aug 8;12(8):e0182433. doi: 10.1371/journal.pone.0182433 (PMC5549724; doi:10.1371/journal.pone.0182433)
Supplement: S1 Fig — (A) XIAP-AS1 expression levels, down-load from TCGA, in stomach adenocarcinoma (n = 285) and normal tissues (n = 33) were analyzed. (B) The survival probability of patients with stomach adenocarcinoma with XIAP-AS1 high (n = 76) or low (n = 209) expression were analyzed. (DOCX) [file pone.0182433.s001.docx]

**S1 Fig**


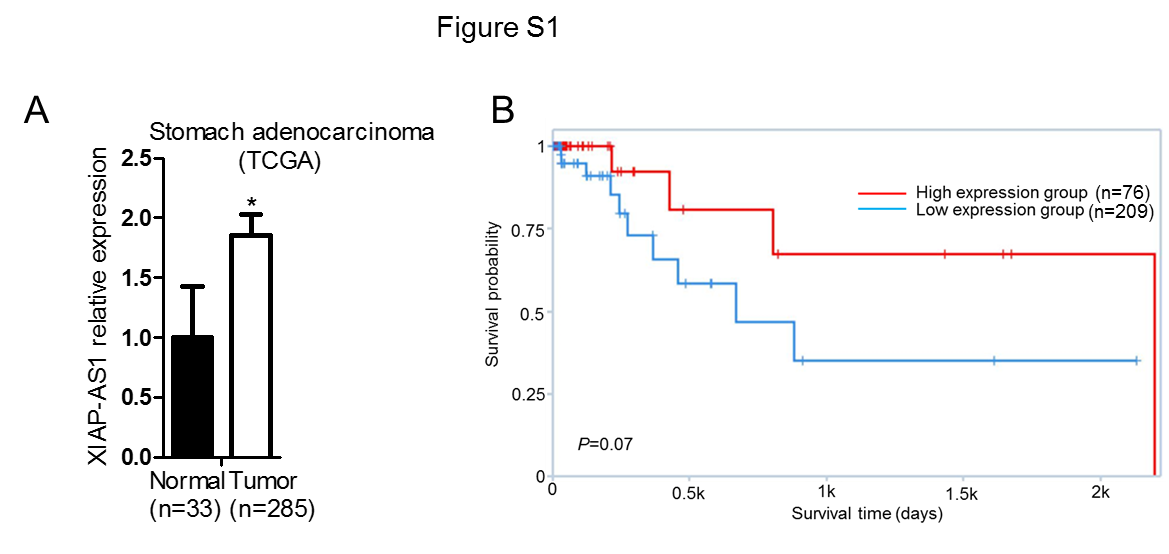


**S1 Fig. XIAP-AS1 expression levels and the survival probability of patients with stomach adenocarcinoma were analyzed.** **(A)** XIAP-AS1 expression levels, down-load from TCGA, in stomach adenocarcinoma (n=285) and normal tissues (n=33) were analyzed. **(B)** The survival probability of patients with stomach adenocarcinoma with XIAP-AS1 high (n=76) or low (n=209) expression were analyzed.
