# Supplementary material for: The long noncoding RNA XIAP-AS1 promotes XIAP transcription by XIAP-AS1 interacting with Sp1 in gastric cancer cells
Source: PLoS One. 2017 Aug 8;12(8):e0182433. doi: 10.1371/journal.pone.0182433 (PMC5549724; doi:10.1371/journal.pone.0182433)
Supplement: S2 Fig — (A) BGC823 shRNA-XIAP-AS1 or shScramble cells were subjected to no TRAIL or TRAIL treatment at a final concentration 100 ng/ml for 24 h and then the percent of apoptotic cells was determined by flow cytometry for Annexin V staining. (B) MNK28 cells were treated without or with TRAIL or the cells were transfected with the XIAP-AS1 expression or empty vector and subsequently treated with TRAIL for 24 h, then the percentage of apoptotic cells was determined using flow cytometry for Annexin V staining. (DOCX) [file pone.0182433.s002.docx]

**S2 Fig**


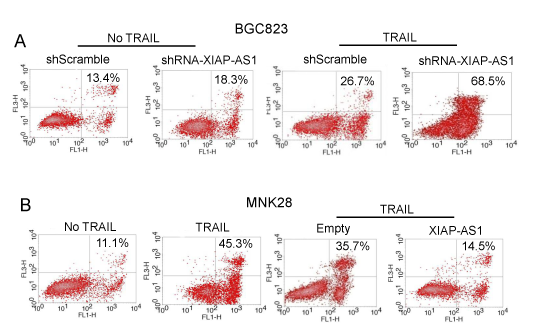


**S2 Fig. XIAP-AS1 knockdown promotes TRAIL-induced apoptosis, whereas XIAP-AS1 up-regulation inhibits the apoptosis induced by TRAIL.** **(A)** BGC823 shRNA-XIAP-AS1 or shScramble cells were subjected to no TRAIL or TRAIL treatment at a final concentration 100 ng/ml for 24 h and then the percent of apoptotic cells was determined by flow cytometry for Annexin V staining. **(B)** MNK28 cells were treated without or with TRAIL or the cells were transfected with the XIAP-AS1 expression or empty vector and subsequently treated with TRAIL for 24 h, then the percentage of apoptotic cells was determined using flow cytometry for Annexin V staining.
